# Biopsychosocial rehabilitation therapy in small fiber neuropathy: research protocol to study the effect of rehabilitation treatment

**DOI:** 10.3389/fneur.2024.1493326

**Published:** 2024-11-13

**Authors:** Aysun Damci, Marlies den Hollander, Janneke G. J. Hoeijmakers, Catharina G. Faber, Mariëlle E. J. B. Goossens, Jeanine A. M. C. F. Verbunt

**Affiliations:** ^1^Mental Health and Neuroscience Research Institute, Maastricht University, Maastricht, Netherlands; ^2^Department of Neurology, Maastricht University Medical Centre, Maastricht, Netherlands; ^3^Adelante Centre of Expertise in Rehabilitation and Audiology, Hoensbroek, Netherlands; ^4^Care and Public Health Research Institute, Maastricht University, Maastricht, Netherlands; ^5^Department of Rehabilitation Medicine, Maastricht University, Maastricht, Netherlands

**Keywords:** controlled clinical trial, rehabilitation, single case study, neuropathic pain, rehabilitation therapy

## Abstract

**Background:**

Small fiber neuropathy (SFN) is a chronic neuropathic pain condition that can lead to a decreased quality of life (QOL) and disability. Current pain treatment is mainly symptomatic, consisting of analgesics, with often disappointing results. There is a need for new, more effective treatment modality. Treatment based on a biopsychosocial approach on SFN-related pain may be a promising alternative. A rehabilitation treatment study protocol is presented with the following main objective: to test the effect of a tailored interdisciplinary rehabilitation treatment targeting both cognitive and psychological factors related to pain, in decreasing disability, and improving QOL in SFN.

**Methods:**

Single-case experimental design. Ten participants with SFN will be included. Every patient will be offered a personalized program based on one of three rehabilitation treatment modules (graded activity, exposure *in vivo* or acceptance and commitment therapy) depending on the most prominent factor maintaining disability. Treatment will be provided for at least 8 weeks with 2 sessions a week.

**Discussion/conclusion:**

This is the first study investigating personalized rehabilitation treatment in patients with idiopathic SFN. The findings are expected to result in an effective treatment for SFN with an increase in QOL and a decrease in disability.

**Clinical trial registration:**

ClinicalTrials.gov, identifier NCT05798949.

## 1 Introduction

Small fiber neuropathy (SFN) is caused by damage to the myelinated Aδ- and unmyelinated C-fibers, leading to chronic, neuropathic pain and autonomic dysfunction (1). In SFN, both physical- and psychosocial domains of quality life (QOL) and physical ability are reduced (2). Biopsychosocial factors including, anxiety, depression, and catastrophic thoughts are observed in SFN (3, 4), which could be effectively treated by biopsychosocial (rehabilitation) treatments (5–7). Reduction of disability and improvement of QOL in various chronic pain conditions have been observed (5–7). Certain treatment modalities have not been investigated in SFN, however, the current pain treatment is mainly based on (neuropathic) pain relief, independent of an underlying cause (8). In SFN, in 47% of the cases an underlying cause is present (9). A pain reduction of 30–40%, at most, has been observed with the most potent analgesia, often with several side effects (8, 10). In SFN, patients experience a lower QOL, when compared to healthy individuals (2). Also, the burden of each patient differs, with special needs adapted to their personal lives (4). Each patient has different complaints with a different degree of severity. Patients also have a unique and personalized coping mechanism to handle their complaints. Therefore, an adequate and personalized treatment of pain, focusing on different aspects, in SFN is an unmet medical need, requiring the search for other possible treatments.

In chronic pain, biopsychosocial factors affect the course and severity of pain intensity and physical disability, indirectly resulting in decreased QOL (11–21). Biopsychosocial factors, including pain catastrophizing, pain-related fear, avoidance behaviour, and depressive mood, determine the extent of experienced disability, independent of pain intensity (22). First, pain catastrophizing is defined as an exaggerated negative mental appraisal of an actual pain experience (23, 24). In the presence of pain catastrophizing, the level of disability seems to be higher (23–27). A second factor is pain-related fear (28, 29). Fear is more disabling than the pain itself (30–32): when activities are seen as a predictor for pain or other negative consequences such as harm or inability, subsequent avoidance- and safety behavior will arise, leading to a reduction of and limitations in activity performance (33). The last factor is depressive mood. Physical disability is more prevalent in the presence of a depressive disorder when compared to situations with the absence of depressive disorder (34, 35). In chronic pain, the effect of disabling psychosocial factors can be reduced by different cognitive behavioral treatment modalities (5, 36–38).

One of these treatment modalities is graded activity (GA), which is effective in improving disability, influencing fear and pain catastrophizing in chronic pain disorders (36, 39). GA aims to improve functional ability step-wise using operant conditioning principles (5, 36). Second, exposure *in vivo* (EXP) aims to change catastrophic misunderstanding of complaints, characterized by an inhibitory learning approach (40). In patients with elevated levels of pain-related fear (chronic low back pain and complex regional pain syndrome type 1), favorable effects of EXP have been reported in diminishing catastrophizing, lowering pain-related fear, improving disability and QOL (5, 6, 33, 37, 41). At last, acceptance and commitment therapy (ACT) aims to increase psychological flexibility rather than changing thoughts and mood, according to the relational frame theory (42–44). ACT is focusing on acceptance and behavioral aspects (42–44). A recent meta-analysis showed evidence to support the use of ACT in the treatment of chronic pain, resulting in the reduction of disability and improvement of QOL (35, 38, 45–47). These three treatment modalities, each with an individual focus on reducing the negative effect of biopsychosocial factors, might also be suitable to improve disability and QOL in chronic neuropathic pain with a somatic underlying cause, such as SFN. A personalized treatment module with three suggested treatment options may be more suitable, focusing on the maintaining factors of each patient (4).

To date, the current available treatments for SFN are generally inadequate. A tailored rehabilitation treatment, targeting the most important maintaining factors of disability and reduced QOL in a specific patient, might be effective because each patient with SFN is unique. Therefore, the main objective of this study is to test the effect of tailored interdisciplinary rehabilitation treatment, targeting the specific psychological and behavioral factors related to pain, in decreasing disability, and improving QOL in SFN.

## 2 Methods

### 2.1 Study design

**Figure 1:**
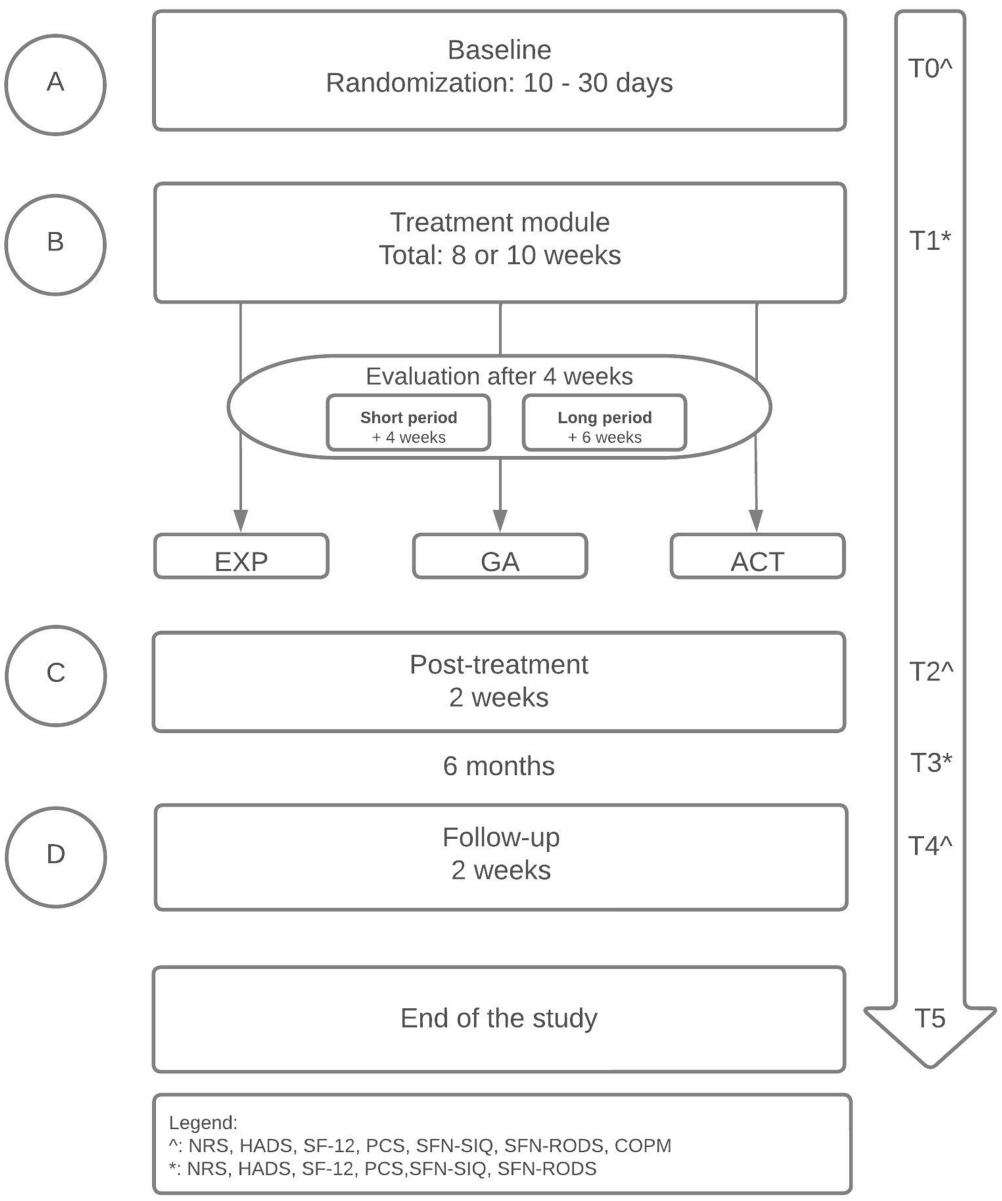
Study flowchart.

The design is a randomized replicated sequential single-case experimental ABCD design (SCED) with multiple measurements (48) (see Figure 1). Each participant will enrol in the baseline period, completing daily measurements with an electronic diary (A). The randomization in the baseline period will define the length of the baseline period (ranging between 10 to 30 days), increasing the internal validity and credibility (49, 50). By the randomization, the start point of the treatment module (date) will be determined (B). Therefore, the start point differs among participants (48, 51, 52). The treatment module will have a duration of 8 or 10 weeks, independent of the treatment type (these logistics are a standard clinical procedure). Each participant will be evaluated by the treatment team according to their personalized goals after 4 weeks. The further treatment module will exist of a short- (4 weeks) or long (6 weeks) treatment module, which will be added to the previous 4 weeks. The content of the short- and long treatment module will not differ. The only difference is the duration between both treatment modules. The differences between the short- and long treatment module will not be analyzed, because that is not the main focus of this study. After the treatment module, a post-treatment of two weeks will follow (C). At last, the study will end with a period two weeks of follow-up (D). Before the start of each period, participants will complete a couple of questionnaires (see Non-daily measures). The length of the baseline period (A) will be randomized between 10–30 days and participants will complete daily measurements with an electronic diary in the baseline period (A), for 8–10 weeks in the treatment module (B), two weeks post-treatment (C) and two weeks at follow-up (D) (see Figure 1, see Daily measures). Before the start of each period, participants will complete a couple of questionnaires (see Non-daily measures). Randomization of the baseline period will occur according to the length of the baseline period (between 10 and 30 days) and the start of the treatment module (B), increasing the internal validity and credibility (49, 50).

This study will be embedded in Maastricht University Medical Centre+ (Maastricht UMC+) at the department of neurology and in Adelante Zorggroep location Maastricht MUMC+. The department of neurology is an expert centre in diagnosing and treating patients with SFN. The diagnosis is made according to the international Besta criteria: typical SFN symptoms and signs, combined with a decreased intraepidermal nerve fiber density in skin biopsy and/or abnormal temperature thresholds in quantitative sensory testing (53). The department of rehabilitation medicine has gained a lot of experience in developing rehabilitation treatments based on cognitive behavioral principles for chronic pain patients (6, 54).

### 2.2 Treatment protocol

The existing treatment protocols for chronic pain conditions were made applicable for patients with SFN, older than 18 years, who are experiencing disability and pain in their daily life. The following exclusion criteria are applicable: 1. the presence of other chronic pain condition than SFN, that may cause pain in the feet and/or damage to the peripheral nervous system and influence QOL, and 2. insufficient comprehension of the Dutch language.

The treatment team will consist of a rehabilitation physician, psychologist, occupational therapist and/or physiotherapist. All team members are experienced in chronic pain rehabilitation, and more specifically in providing the three modalities GA, EXP and ACT. The same rehabilitation team will be involved in the different treatment modules that will be offered (see later). The researchers are not involved in the treatment procedures; however, they will have access to the daily measurements to keep track of completion.

The treatment protocol is divided into three parts: (1) intake by a rehabilitation physician, (2) interdisciplinary screening, and (3) treatment modules.

#### 2.2.1 Intake by a rehabilitation physician

After referral by the neurologist, the rehabilitation physician will invite every participant to a first consultation (T0) (Figure 1). During this consultation, a subjective examination and physical examination will take place. The rehabilitation physician will determine if there is an indication for interdisciplinary rehabilitation. In addition, the physician will assist the patient in setting goals for treatment. It is important that a patient has functional goals to achieve in daily life, and is not just aiming to reduce pain. The patient will be screened according to the eligibility criteria of the study. The participants have to give written informed consent. The rehabilitation physician has the final responsibility for the participants during the study period.

#### 2.2.2 Screening

During the interdisciplinary screening, the patient’s personal situation (i.e., disabling-, biomedical-, psychological-, and social factors) is thoroughly evaluated with the aim to identify the best personal module for every individual patient. A personalized treatment plan will be based on one of three treatment modules: GA, EXP, and ACT. All modules are provided by an interdisciplinary team. Each team member has a specific role in the team; however, all team members aim to analyze the underlying reason for the experienced disability and to assist the patient to set treatment goals. The best module fit is dependent on the clinical impression of the patients. This means that the team members of an interdisciplinary team will evaluate each patient individually. After their evaluation, they will discuss in a team meeting their individual findings, and discuss which treatment module is most suitable for each patient. Each treatment module has a different treatment accent. Exposure of vivo is suggested in patients who experience fear of movement or pain (5). Graded activity will be suggested in patients with a low load capacity to gradually increase their activity tolerance (6, 7). Acceptance and commitment therapy is suggested in patients who are stuck in rule-governed behavior and struggle with their disease or (pain) complaints (8).

There are differences between team members:

- The psychologist will analyze the impact of cognitive (cognitions about pain and activities, threat beliefs), emotional (pain-related fear, mood), psychosocial (expectations about treatment, history of (psychological) factors, life events, dominant life rules), and behavior (coping, pain-behavior) on the participants with SFN.
- The occupational therapist will analyze the impact on current and former daily activities and the underlying motives for persistence, avoidance or safety behavior. With the Canadian Occupational Performance Measure (COPM), the most prominent problematic activities will be identified and scored on performance and satisfaction (55). The activities might serve as goals during treatment.
- The physiotherapist will observe a participant during activities to analyze performance, disability and disability-related factors (such as fear or pain-contingent functioning).
- A selection of 10–15 pictures of the Photographic Series of Daily Activities (PHODA) is used to assess (negative) expectations and beliefs a patient might have about performing activities presented in the pictures (56).

Subsequently, there will be a plenary meeting of all team members (including the rehabilitation physician) to discuss their findings (in absence of the participant). During this meeting, an overall conclusion about the factors maintaining disability will be set, and the treatment module that bests fits will be identified (see later). This choice is based on the most prominent maintaining factor for physical disability in this specific patient as observed by the team.

Thereafter, an educational session with the participant will follow. The educational session will be elaborated on by the rehabilitation physician and the psychologist. In this educational session, the rehabilitation physician will discuss the aetiology of SFN and explain the physiology of pain and the pathophysiology of pain and SFN. Thereafter, the hypothesized mechanism for pain-related disability and chronic pain maintenance for this individual patient will be introduced and discussed with the patient (57–59). The psychologist will provide a patient-specific explanation of the treatment rationale of, respectively, GA, EXP or ACT (57, 58). The educational session aims to acknowledge the patients’ complaints and provide a patient with the viewpoint that it is possible to take control back over one’s functioning (instead of directly trying to control one’s pain), either by a time-contingent performance of activities (GA), challenging negative beliefs about the consequences of activities (EXP), increasing psychological flexibility and take value-based actions (ACT). The educational session will last for a maximum of 60 min and will be individually provided.

During the screening, the treatment team will decide if a participant is suitable for one of the three treatment modalities. If a participant is suitable, additional information will be sent to the research team, including baseline characteristics (name, date of birth, gender), treatment modality choice, and personal treatment goal. This personalized treatment goal will be embedded in the daily dairy to measure the influence of the treatment modality on the personalized goal(s). The treatment modality will be individually planned for each participant. However, the length of the baseline period will be randomized, see section “Study design,” and is not dependent of the treatment team nor the treatment modality.

#### 2.2.3 Treatment

Each treatment module will be provided by a treatment team consisting of the rehabilitation physician, a psychologist, and an occupational therapist or a physiotherapist, all of whom are experienced in each treatment module. One of the three treatment modalities will be chosen. Participants have to complete the treatment module to which they are allocated. Participants will only follow one treatment module. The treatment will continue for 8–10 weeks, with a twice a week a 1-h session. The duration will be determined by team members and the rehabilitation physician after the first phase of 4 weeks. During a team meeting, the team members will consider a short or long phase-2 based on the progress in the first phase (see Table 1).

**Table 1:** Overview and summary of the treatment modalities.

	Graded activity	Exposure *in vivo*	Acceptance and commitment therapy
Team meeting	Meeting 1: 4 weeks after start of the treatment module Meeting 2: the final treatment session Meeting 3: 6 weeks after the completion		
Duration (total)	8–10 weeks		
Treatment schedule	Phase 1: 8 sessions in total with 1-h/session According the progress in phase 1, the team members choose the duration of phase-2 (short or long) Phase 2-short: 8 sessions in total with 1-h/session Phase 2-long: 12 sessions in total with 1-h/session		
Therapist	Psychologist and physical- or occupational therapist		
Underlying paradigm	Operant conditioning	Inhibitory learning approach (classical conditioning)	Relational frame theory
Treatment aim	Increase activity tolerance and healthy behaviour	Modifying fear-related beliefs resulting in disability/restrictions	Increase psychological flexibility
Scientific evidence	Reduction of disability, pain catastrophizing and/or pain intensity in chronic low back pain and chronic neuropathic pain (39)	Reduction of disability and pain catastrophizing in complex regional pain syndrome type 1 and chronic back pain (5, 6)	Reduction of disability in chronic pain disorders (46, 47)
Selection based on	COPM	Outcome of PHODA	COPM

COPM; Canadian occupational performance measure; PHODA, photographic series of daily activities.

##### 2.2.3.1 The procedure of GA

GA aims to influence activity tolerance and healthy behavior with principles of operant conditioning (5, 36). First, the patient’s (pain-contingent) baseline tolerance for a specific activity is assessed. Second, a time-contingent schedule will be developed increasing the activity stepwise to the personal treatment goals of the patient. Initially, the treatment activities in the time-contingent phase have to be performed at 70–80% of the baseline activity level. Throughout the treatment sessions, the activity and the tolerance level will increase due to positive reinforcement (5, 60), enabling a patient to change functioning from pain-contingent (via time-contingent) to goal-contingent.

##### 2.2.3.2 The procedure of exposure in vivo

Exposure *in vivo* is the treatment of choice if the participant has negative expectations about performing activities or if the participant is experiencing fear of pain or movements (5, 6, 61). The main principle of exposure *in vivo* is based on the principles of the inhibitory learning approach (40). With the aid of the Photograph Series of Daily activities (PHODA), it is possible to assess (negative) expectations a patient has about performing activities. These expectations will be challenged with behavioral experiments, providing a mismatch between the patient’s expectations and what actually happens (62). These new experiences will result in the inhibition of the old, fearful memory pathways, enabling patients to end avoidance and safety behavior (40, 63).

##### 2.2.3.3 The procedure of ACT

The main purpose of ACT is to increase psychological flexibility, which is defined as the ability to make contact with one’s experience in the present moment, and, based on what is possible at that moment, choosing behavior in the pursuit of goals and values (64). ACT uses an acceptance-based approach to manage internal experiences, instead of correcting cognitive errors or changing physical experiences with six core principles; contact with the present moment, values, committed action, self as context, defusion and acceptance (42, 59). Each core principle is based on the relational frame theory (44). Throughout several treatment sessions, each core principle will be featured (42).

### 2.3 Team meetings and aftercare

During treatment, at least two team meetings will take place to evaluate the individual treatment progression on the treatment goals and the patient’s general functioning in daily life activities. In the final treatment session, the participant evaluates his/her progression in the performance of daily life activities and other possible treatment effects with the team members. Furthermore, a personalized relapse prevention plan is made. Six weeks after program completion, the patient will revisit the team to evaluate current functioning in daily life situations. The COPM will be re-scored. Six weeks thereafter, a final consultation with the physician will take place. The data of the two team meetings will not be shared with the research group. The research group will have the SCED data to be able to draw conclusions about the effectiveness and changes of the patient. During treatment, no one (researchers nor clinicians) will have access to these data, so the in clinical practice regular evaluating moments are used to describe the progress of the patient in clinical terms. If a treatment module is considered not suitable for a patient along the way, and this patient has to change to another treatment module, the research group will be notified of this change.

### 2.4 Measurement and outcome

The main study endpoints are disability, measured with the Pain Disability Index (PDI) and SFN-Rasch-built Overall Disability Scale (SFN-RODS), and health-related QOL, measured with the 12-item Short Form Survey (SF-12). Secondary endpoints are pain intensity, catastrophizing, mood (anxiety and depression) and SFN-related complaints, measured with Numeric Rating Scale (NRS), Pain Catastrophizing Scale (PCS), Hospital Anxiety and Depression Scale (HADS), and SFN-Symptom Inventory Questionnaire (SFN-SIQ). All the endpoints will be measured at several measurement points with daily and non-daily measurements (see Figure 1). The primary endpoint is based on the clinical improvement of the participants.

### 2.5 Daily measures

To measure the primary and secondary outcomes, participants will complete an electronic diary daily. An e-mail will be sent each day to remind participants to fill in the electronic diary. The diary is consisting of 9 questions about pain intensity, disability and physical activity, QOL and anxiety and depression. Table 2 shows an example of the diary questions.

**Table 2:** Overview of diary questions.

Pain intensity	How was your average pain during the day?
	How was your maximal pain during the day?
Pain diversity	How well did you succeed to handle your pain today?
Mood	If I have pain, I keep thinking how much I want the pain to stop
	I feel cheerful
	I score my life today
Activity	How hard was it (or would It be) to perform this activity?

Each question could be answered on a Likert scale 11 (0–10), in which 0 stand for not at all, and 10 for the most.

### 2.6 Non-daily measures

Disability. Disability will be measured with two questionnaires, PDI and SFN-RODS. The PDI investigates the level of disability in painful situations. The questionnaire consists of 7 questions. Each question can be answered on a Likert-11-scale (65). A higher score indicates more disability in daily life. The SFN-RODS is measuring daily activities and consists of 32 questions with each 3 answer options (66).

Quality of Life. SF-12 questionnaire inventories global health with 8 questions (67). Each question has 5 answer options. A lower score on the SF-12 is indicating a worse health status.

Pain intensity. NRS will measure the pain intensity, ranging from 0 to 10. A higher score is indicating more pain complaints (68).

Anxiety and depression. The HADS questionnaire measures depressive and anxious symptoms (69). The questionnaire is consisting of 14 questions, each of 7 questions related to depression or anxiety. The score can be divided into several scales: “normal” between 0 and 7, “borderline” between 8 and 10, and “abnormal” higher than 11 (70). A higher score is indicating the presence of anxiety and/or depressive symptoms.

Pain catastrophizing. With PCS, the level of pain-catastrophizing can be measured. The questionnaire consists of 13 questions. A higher score is indicating the presence of catastrophic thoughts (71).

SFN-related complaints. The SFN-related questionnaires are reliable and developed to measure SFN-specific complaints. The SFN-SIQ is a questionnaire to measure autonomic dysfunction and pain in SFN with 14 questions (66). Four different answer options are available. The centile metric total scores can be calculated, with a score between 0 and 100. A higher score on the SFN-SIQ is indicating the presence of more SFN-related complaints.

Rehabilitation goals. The COPM is an evidence-based outcome measure designed to measure the client’s self-perception of performance in everyday living over time. In a semi-structured interview, the client identifies problem areas in daily functioning and rates performance and satisfaction with performance. Two mean scores, for performance and satisfaction, will be obtained.

All the mentioned questionnaires are reliable and validated to use in chronic pain conditions (65, 66, 72–77).

### 2.7 Data collection

Data of age, sex, duration of SFN, use of pain medication, pain intensity, disability, and SFN-related diagnostic (such as skin biopsy outcome and outcome of quantitative sensory testing) outcomes will be collected. Only principal investigator and the coordinating researcher will have access to the data.

The study protocol is reported in accordance with the SPIRIT guidelines [Standard Protocol items: Recommendations for Interventional Trials (78)].

### 2.8 Data management

All data will be entered into a web-based trial management system by members of the research team. All data will be coded with unique study number. The research team will have a list showing codes and patients’ names, separately from the study database. Only the study coordinator will have access to this list, and the main database.

#### 2.8.1 Amendments

Amendments, or changes of the research protocol after IRB approval, will be reported to the METC that approved the research protocol.

### 2.9 Sample size calculation

Based on previous experience with single-case experimental designs in chronic pain, 10 participants provide sufficient power to answer our hypothesis that rehabilitation based on biopsychosocial principles is effective to decrease disability and improving QOL (79). Therefore, at least 10 participants need to complete the study. In case a participant withdraws, a new participant will be included. Studies that tested the statistical properties of the randomization test used in this type of design showed that type I error probability of the randomization test was maintained at an acceptable level (48, 80, 81). SCED studies have multiple measurements, resulting in a large effect size and great power (82). Each participant is equal to one experiment. And each experiment will be repeated at least for 10 times. The variation of the treatment starting point will show if a treatment is effective, and that the significant outcome is related to the treatment modality. With a kind of meta-analysis, it is possible to combine all the *p*-values (of each participant). Therefore, this study design is a powerful method due to the multiple measurements and the number of participants (10 repetitions). Compared to RCT studies, more than 100 participants will be included, with only a couple of measurements per participant. Contrary to SCEDs, daily and multiple measurements will be gathered, and therefore, a sample size of 10 is enough to analyze the data with a sufficient outcome. The total number of participants and permutations is not related to the power of the design, however, calculation of the p-value is possible by dividing the total number of permutations (83). The aim of this study is to observe if a multidisciplinary rehabilitation treatment modality is suitable and effective in the treatment of psychosocial factors in SFN. certain treatment modalities are used as care as usual in different chronic pain conditions, in which there is always a selection between different treatment options.

### 2.10 Statistical analysis

The baseline characteristics will be presented in means ± standard deviation, medians (interquartile range) or percentages, as appropriate, including age, sex, duration of SFN, use of pain medication, pain intensity, disability, and SFN-related diagnostic (such as skin biopsy outcome and outcome of quantitative sensory testing). The outcome of the questionnaires will be analyzed using SPSS (Version 27). Normality assumptions will be checked with the Shapiro–Wilk test. Normally distributed treatment effects variables will be analyzed with the paired student’s t-test. Non-normally distributed treatment effects variable will be analyzed with the paired Wilcoxon test.

The analysis of the daily dairy data will be conducted with the Shiny SCDA, which is a web application to analyze SCED studies (51, 84). Shiny SCDA, a web-based application, has been developed to easily analyze and investigate intervention effects of the SCED data at an individual level, but also to combine the SCED data through multilevel modeling and randomization tests, which create a higher internal validity (48). Randomization test are based on the random determination of the moments of phase change points to test a null hypothesis about treatment effects (85). Randomization tests have the advantage of being valid for single-case experiments without making distributional assumptions, of being easy to apply and of being extremely versatile for even the most complex single-case design.

Each SCED will be individually analyzed, but we will use *p*-value combining (of the 10 SCEDs in this project), which has the advantages that it is broadly applicable and that it is distribution-free without converting the scores to ranks or signs. The following schedule will be used to investigate when treatment effects occur during the study period:

- The baseline data (phase A) will be compared with the intervention, post-intervention and follow-up data (phase B + C + D).
- The intervention data (phase B) will be compared with the baseline data (phase B) and with the post-intervention and the follow-up data (phase C + D).
- The post-intervention data (phase C) will be compared with the baseline data (phase A) and with the follow-up data (phase D).
- The follow-up data will be compared with the baseline data (phase A) and the intervention data (phase B).

A *p*-value of 0.05 will be set.

It is important that the various periods [ea. (A), (B), (C), (D)] show a substantial amount of non-overlapping data. Nonoverlapping data of pairs (NAP) is an indicator of differences between the different phases in the treatment schedule. We do not expect that there will be an immediate effect from the start of treatment, however, we expect that there will be a gradual positive effect.

## 3 Discussion

In this study, the effect of rehabilitation treatment in patients with idiopathic SFN will be studied. Although the effect of rehabilitation interventions has already been studied in several chronic (neuropathic) pain conditions, this is not the case for SFN. The lack of rehabilitation treatment in SFN is remarkable because the mechanism of pain in several chronic neuropathic pain conditions with also a somatic underlying component seems comparable (6, 86). This is the first study to investigate the efficacy of a personalized intervention based on the principles of exposure *in vivo*, GA and ACT in SFN to the best of our knowledge. Therefore, this study is distinguishing itself by aiming to modify patterns interfering with daily activities and important life roles.

There are some strengths and limitations. A strength of this study could be the study design. With a limited sample size, SCEDs allow conducting reliable and valid studies due to the repeated measurements. Whereas randomized controlled trials are based on a few measurement moments in many subjects, SCEDs are characterized by many measurements in a few subjects (87). The design results in a high internal and external validity (49, 88). Another strength could be that the effect of rehabilitation (using cognitive behavioral principles) is not known in SFN. To date, the efficacy of rehabilitation in SFN has not been investigated. However, impressive outcomes have been reported about the impact of comparable rehabilitation treatment modalities in other neuropathic pain conditions like complex regional pain syndrome type 1 and painful diabetic neuropathy comparable with SFN (6, 37, 79). A limitation of this study could be the fact that the intervention in this study is personalized and thus can comprise various modalities, which could, however, also be a strength. The length of these modalities will also differ between the participants, but this is common in rehabilitation: rehabilitation teams are specialized in examining and identifying the maintaining factors of (chronic) pain and disability. After all, SCED will enable us to conclude with insights into the effect of tailored rehabilitation treatment in SFN.

In conclusion, this is the first study to investigate the efficacy of rehabilitation intervention in patients with SFN. Such a rehabilitation intervention may become a new treatment option with the aim to increase QOL, decreasing disability and reducing pain intensity in idiopathic SFN.
